# Identification and characterization of novel lineage 1 Powassan virus strains in New York State

**DOI:** 10.1080/22221751.2022.2155585

**Published:** 2022-12-20

**Authors:** Rachel E. Lange, Alan P. Dupuis II, Melissa A. Prusinski, Joseph G. Maffei, Cheri A. Koetzner, Kiet Ngo, Bryon Backenson, JoAnne Oliver, Chantal B.F. Vogels, Nathan D. Grubaugh, Laura D. Kramer, Alexander T. Ciota

**Affiliations:** a Department of Biomedical Sciences, State University of New York University at Albany School of Public Health, Albany, NY, USA; b New York State Department of Health, The Arbovirus Laboratory, Wadsworth Center, Slingerlands, NY, USA; c New York State Department of Health, Bureau of Communicable Disease Control, Vector Ecology Laboratory, Albany, NY, USA; d New York State Department of Health, Bureau of Communicable Disease Control, Syracuse, NY, USA; e Department of Epidemiology of Microbial Diseases, Yale School of Public Health, New Haven, CT, USA

**Keywords:** Powassan virus, deer tick virus, New York State, lineage 1, *Ixodes scapularis*

## Abstract

Powassan virus (POWV, family *Flaviviridae*) is a reemerging tick-borne virus endemic in North America and Russia. In 1997, a POWV-like agent was isolated from *Ixodes scapularis* in New England and determined to be genetically distinct from the original POWV isolate. This revealed the existence of two lineages: lineage 1, prototype Powassan virus (POWV-1) and lineage 2, deer tick virus (DTV). POWV-1 is thought to be primarily maintained in a cycle between *I. cookei* and woodchucks and *I. marxi* and squirrels, while DTV is primarily maintained in a cycle between *I. scapularis* and small mammal hosts. Recent tick, mammalian, and human isolates from New York State (NYS) have been identified as DTV, but for the first time in 45 years, we detected four POWV-1 isolates, including the first reported isolation of POWV-1 from *I. scapularis*. We aimed to investigate genotypic and phenotypic characteristics of recent NYS isolates through sequence analysis and evaluation of replication kinetics *in vitro* and *in vivo*. Our sequencing revealed genetic divergence between NYS POWV-1 isolates, with two distinct foci. We found that POWV-1 isolates displayed variable replication kinetics in nymphal ticks but not in cell culture. POWV-1 isolated from *I. scapularis* displayed increased fitness in experimentally infected *I. scapularis* as compared to historic and recent POWV-1 isolates from *I. cookei*. These data suggest the emergence of divergent POWV-1 strains in alternate tick hosts and maintenance of genetically and phenotypically discrete POWV-1 foci.

## Introduction

Powassan virus (POWV; *Flaviviridae*) is an emerging tick-borne virus of the tickborne encephalitis virus serogroup. POWV was first identified as a human pathogen in 1958 when virus was isolated from the brain tissue of a fatal pediatric encephalitis case [1]. Since then, POWV has been detected in human, mammalian, and tick samples from the United States (US), Canada, and Russia [2]. Additionally, serological evidence suggests the presence of Powassan virus in Mexico [3].

In North America, POWV is comprised of two lineages: lineage 1, prototype Powassan virus (POWV-1) and lineage 2, deer tick virus (DTV) [4–6]. This distinction was first recognized in 1997 from a genetically distinct POWV-like agent isolated from *Ixodes scapularis* collected in New England [7]. Further investigation indicated each lineage is maintained in separate enzootic cycles in nature; with POWV-1 thought to be primarily maintained between *I. cookei* and woodchucks (*Marmota monax*) and *I. marxi* and arboreal squirrels (*Sciuridae spp.*)*,* and DTV primarily maintained between *I. scapularis* and small mammal hosts including white-footed mice, voles, and chipmunks [7–15]. In addition, recent evidence suggests shrews are important reservoir hosts for DTV [16]. *Ixodes spp*. are the primary arthropod host for POWV-1 and DTV, but the virus has also been isolated from *Dermacentor spp*. and *Haemaphysalis spp*. [2, 17, 18].

Despite the existence of two lineages, both POWV-1 and DTV cause human disease with most recent cases in the northeastern US thought to be the result of DTV infections associated with bites from *I. scapularis* [1, 19]. Typically, POWV infections initially present as non-specific, flu-like symptoms including fever, nausea, headache, and fatigue [20–22]. Infection can progress into encephalitis and/or meningitis with hemiplegia, muscle wasting, headaches, and memory problems [20–22]. Difficulty with diagnosis and the lack of therapeutics and vaccines for POWV infection make the increased prevalence and case numbers a notable concern in North America. Infection is reported to have a 10-15% mortality rate and up to 50% of disease survivors exhibit lifelong neurological deficits [21, 22]. There has been an increase in the number of reported POWV encephalitis cases in humans over the past two decades [20, 22–24]. From 1958 to 1998, 27 cases were reported [25]. From 2006 to 2016, 99 cases were reported with ∼90% of those being neuroinvasive, and with 5 states reporting cases for the first time (Connecticut, Minnesota, New Hampshire, Rhode Island, and Virginia) [23]. The upward trend of average yearly cases (0.7 cases/year in 1958-1998 to 10 cases/year in 2006-2016) has continued with an average of 16 cases/year reported between 2011–2020 [24]. The highest number of cases (n = 43) was documented in 2019 with Minnesota, Wisconsin, Massachusetts, Connecticut, and New York State (NYS) reporting the highest case burdens [24]. Factors that likely contributed to this increase include range expansion of *I. scapularis* and concomitant emergence of DTV, as well as heightened awareness, surveillance, and clinical assay development [26–28].

The first case of POWV encephalitis in the US was reported in NYS in 1972 [29]. Since 2003, NYS has reported unprecedented increases in reported human cases that coincide with increased detection of DTV in *I. scapularis*, especially in the Lower Hudson River Valley [15, 20]. High DTV infection rates in *I. scapularis*, serological evidence in mammals, and details of two fatal human cases from that region were reported between 2007–2012 [15, 19, 30]. Despite robust surveillance and clinical programmes in NYS, POWV-1 has not been detected since 1975 [31]. For the first time in 45 years, we detected four POWV-1 positive pools and isolated virus from ticks collected in NYS, including the first isolation of POWV-1 from *I. scapularis.* Genetic and phenotypic characterization of these unique NYS isolates provides insight into the relationships between POWV expansion, diversification and invertebrate host range..

## Methods

### Tick collection

Since 2007, the New York State Department of Health (NYSDOH) has been conducting tick surveillance on public lands across NYS for POWV. Questing ticks were collected by standard drag and flag sampling techniques as described [32, 33]. Briefly, a 1 m^2^ piece of white flannel, corduroy, or similar cloth was dragged over leaf litter and low vegetation targeting nymphal and adult ticks, primarily *I. scapularis*. Ticks were removed from the cloth and placed in 100% ethanol or kept alive at 4°C and 100% humidity until they were identified to species, sorted by developmental stage and sex (adults), and pooled for testing. Pooled ticks were stored at −20 °C if collected in ethanol or fresh-frozen at −80 °C if maintained alive.

In an effort to increase the number of *I. cookei* sampled, during 2020 NYSDOH collaborated with the United States Department of Agriculture (USDA) to test ticks removed from nuisance woodchucks (*Marmota monax*) trapped throughout NYS. Live ticks were removed from euthanized woodchucks in the field, placed in 15 mL conical tubes until identified, sorted, and pooled. Pooled ticks were fresh-frozen and stored at −80 °C until tested.

### Cells and viruses

Baby hamster kidney cells (BHK-21, ATCC, CCL-10) were grown in minimal essential media (MEM, Gibco, Invitrogen Corp, Carlsbad, CA) supplemented with 10% heat inactivated fetal bovine serum (FBS, Hyclone, Logan, UT) and maintained at 37 °C, 5% CO_2_. Confluent monolayers were prepared for infection by seeding six-well tissue culture plates with 1.0 × 10^6^ cells per well and maintained for 3–4 days prior to experimental infection.  

Viral isolates POWV-1 64-7062, POWV-1 46-001, POWV-1 63-002, POWV-1 19065-059, and DTV 18071–054 were identified and collected through NYSDOH tick surveillance and testing (Table 1). POWV-1 and DTV positive pools were identified by a multiplex quantitative reverse transcriptase polymerase chain reaction (qRT-PCR) as previously described [15]. Viral isolates were generated by amplification of qRT-PCR positive tick homogenates on African green monkey kidney cells (Vero, ATCC, CCL-81) or BHK-21 cell (ATCC, CCL-10) culture. Upon observation of cytopathic effect (CPE), supernatant was collected, clarified, and heat inactivated FBS was added to 20% of the total volume.

**Table 1.** Recent and historic POWV-1 and DTV isolates collected in New York State.

Strain	County	Year	Host	Lineage
POWV-1 64–7062	St. Lawrence	1964	*I. cookei*	1
POWV-1 46–001	Jefferson	2018	*I. cookei*	1
POWV-1 63–002	Orange	2018	*I. cookei*	1
POWV-1 19065–059	Oneida	2019	*I. scapularis*	1
DTV 18071–054	Orange	2018	*I. scapularis*	2

### Sequencing

RNA was extracted from qRT-PCR positive POWV-1 or DTV pools using an automated MagMAX nucleic acid extraction kit and associated instrument (ThermoFisher Scientific, Waltham, MA, USA). Full genome sequences were obtained by amplification of ∼2 kb fragments of 6 overlapping POWV primer sets (fragment 1 [5’UTR-2620]: F-AGATTTTCTTGCACGTGTGTGCGG R-TCTCCACATGGCCATTTCAAGTCT, fragment 2 [2116-4792]: F-CAGCAGTGGTTTCAGAAAGGCAGT R-ACGTCTTCACGCACATCCGCCCA, fragment 3 [3107-5581]: F-ACTGCACATGGCCAGCAAGTCACA R-TCACTCACTATGGCTCCTTTGGA, fragment 4 [4423-7197]: F-ATGGGGAACTTGCACTTGACAGAG R-GTGGGCGTCGCTCCTACC fragment 5 [6319-9300]: F-GAACTGGTCACGTTCAGAAGCCC R-TCCATGTACCGAAGGATCTGCTCT, fragment 6 [8566-3’UTR]: F-TGGGGCAGCTATCGCACTC R-AGCGGGTGTTTTTCCGAGTCACAC) using a Superscript III One-Step RT–PCR kit (ThermoFisher Scientific, Waltham, MA, USA). Products were visualized on a 1% agarose gel. Amplicons of each 2 kb fragment were pooled for next generation sequencing (NGS). NGS was carried out using the Illumina MiSeq platform (2 × 500 bp PE, Illumina, San Diego, CA, USA) at the Wadsworth Centre Advanced Genomics Technologies Core.  

Sequence analysis was performed in Geneious Prime 2021.2. Reads were paired, merged, primers trimmed, and mapped to reference POWV-1 (POWV-1 LB, NC003687) and DTV (DTVWiA08, HM440560.1) genomes, respectively. Consensus sequences were generated using a 65% minimum frequency threshold for nucleotide calling. Phylogenetic trees and individual amino acid substitutions were inferred following MAFFT alignments of full genome consensus sequences obtained from our sequencing or available sequences from Genbank [34], (Table S1). Identified consensus level non-synonymous mutations were cross referenced in the publicly available “Powassan-genomics” for confirmation [35]. Trees were generated using the Geneious Tree Builder based on neighbour joining trees, Tamura-Nei distances, and 1000 bootstraps and visualized in Fig Tree version 1.4.4.    

### Plaque assay

Plaque assays were performed using confluent monolayers of BHK-21 cells in six-well tissue culture plates (STEMCELL Technologies, Seattle, WA). Cells were inoculated with 0.1 mL of sample in duplicate and allowed to adsorb for 1 h at 37 °C, 5% CO_2_ with rocking every 15 min. After adsorption, an overlay of MEM, 10% heat inactivated FBS, and 0.6% oxoid agar was added to each well. Plates were incubated with the first overlay for 3 days at 37 °C, 5% CO_2_ before addition of a second overlay consisting of MEM, 2% FBS, 0.6% oxoid agar, and 2% neutral red (Sigma-Aldrich Co., St. Louis, MO, USA). Plates were incubated overnight at 3 °C, 5% CO_2_ and plaques were counted the following day. Viral titres are expressed as plaque-forming units (PFU) per millilitre.  

### In vitro growth kinetics

Viral growth kinetics were carried out in six-well tissue culture plates with a confluent monolayer of BHK-21. Cell monolayers were inoculated in triplicate with 0.1 mL of inoculum at a multiplicity of infection (MOI) of 0.01 PFU/mL and allowed to adsorb for 1 h at 37 °C, 5% CO_2_. After adsorption, inoculum was removed, and wells were washed three times with 2.0 mL of phosphate-buffered saline (PBS). After washing, 3.0 mL of MEM supplemented with 10% heat inactivated FBS was added to the wells. At 1, 24, 48, 72, 96, and 120 h post infection (HPI) 0.1 mL of supernatant was collected. All samples were diluted (1:10) in BA-1 media (M199 medium with Hank’s salts, 1% bovine albumin, TRIS base (tris [hydroxymethyl] aminomethane), sodium bicarbonate, 20% FBS, and antibiotics) and stored at −80 °C. Viral load was determined by plaque titration on BHK-21 cells and replication curves were generated based on mean titre for each timepoint. All samples were analyzed using a two-way analysis of variance (ANOVA) with Tukey’s multiple comparisons post hoc tests. All statistics and graphs were generated in GraphPad Prism version 9.0.1. 

### Synchronous infection of ticks

The following reagent was provided by Centres for Disease Control and Prevention for distribution by BEI Resources, NIAID, NIH: *Ixodes scapularis* Nymph (Live), NR-44116. Ticks were maintained at 95% relative humidity (RH) and 20 °C on a 16:8 light:dark cycle. *I. scapularis* nymphs were infected as described previously [36, 37]. Nymphs were subject to reduced RH (65%) for 48-72 h prior to infection. After reduced RH pretreatment, nymphs were suspended in 1 × 10^6^ PFU/mL of POWV-1 or DTV diluted in BA-1 and incubated at 34 °C for 1 h. Tubes were checked every 15 min to ensure nymphs were immersed in viral suspension. After incubation, nymphs were chilled on ice, washed twice with PBS, and individually dried to remove excess moisture. Nymphs were moved to Plaster of Paris jars and stored at 95% RH and 20 °C until collection and processing.  At 7-, 14-, 21-, and 28-days post infection (DPI), infected ticks were moved to individual tubes with 5 mm stainless steel BBs (Daisy Outdoor Products, Roger, AR, USA) and placed at −70 °C until processing.

### Viral detection in experimentally infected ticks

Whole nymphs were homogenized in 0.6 mL of diluent (20% heat inactivated FBS in Dulbecco PBS, 50 ug/mL penicillin/streptomycin, 50 ug/mL gentamicin, and 2 ug/mL fungizone [Sigma-Aldrich, St. Louis, MO, USA]) using a Retsch Mixer Mill, MM 301 (Retsch Inc., Newtown, PA, USA) at 30 cycles/second for a 4-minute cycle. RNA was extracted from homogenates using an automated MagMAX nucleic acid extraction kit and associated instrument (ThermoFisher Scientific, Waltham, MA, USA). Viral detection was carried out by a multiplex POWV-1/DTV qRT-PCR assay [15]. Relative PFU/mL was determined by a standard curve generated based on ten-fold dilutions of a known viral titre of the respective isolate being tested. Two separate experiments were conducted and viral titres from individual nymphs were compared at each timepoint using a one sample student’s t-test. No samples showed significant differences, so experiments were combined for further analysis. All samples were then analyzed using a two-way analysis of variance (ANOVA) with Tukey’s multiple comparisons post hoc tests. All statistics and graphs were generated in GraphPad Prism version 9.0.1. 

## Results

### Tick surveillance

**Figure 1.**
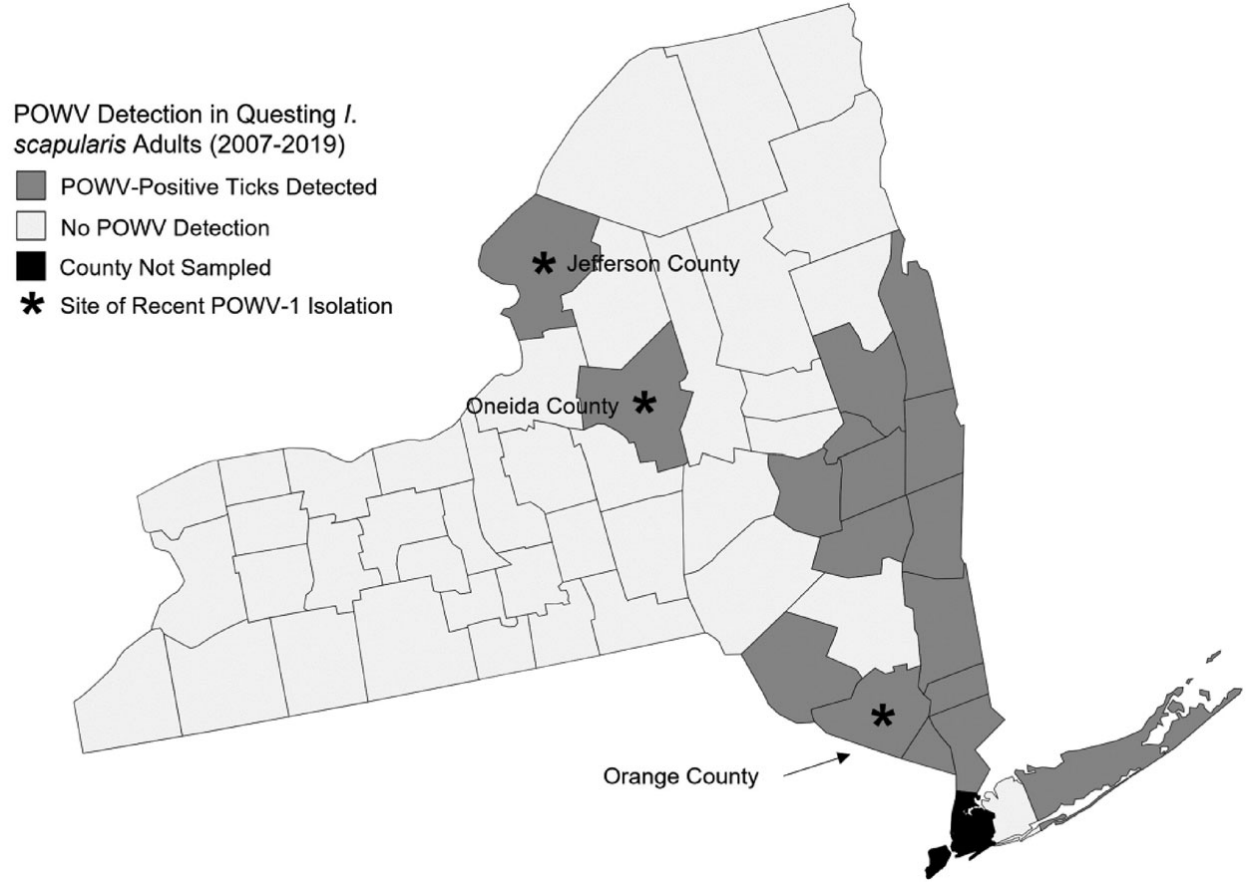
Powassan virus (POWV-1) and deer tick virus (DTV) surveillance across New York State (NYS) between 2007-2019. All counties are sampled yearly for questing adult *Ixodes scapularis* and tested for POWV except for 4 counties outside of New York City. Counties shaded in gray represent those in which at least 1 tick has been positive for POWV and those in white represent no viral detection. Counties with stars indicate regions which recent NYS POWV-1 isolates were collected. Stars do not represent location within the county of the collection site and were placed concentrically.

During 2007-2019, NYSDOH collected and tested for POWV in more than 61,900 questing *I. scapularis* from 53 of 57 counties outside of New York City. From these ticks, POWV was identified in 183 pools. All but two positive pools were identified as DTV. Two strains, 19065–058 and 19065-059, were identified as POWV-1. Both strains were isolated from adult *I. scapularis* pools, 1 male and female each (n = 8), collected in 2019 from Oneida County, NY (Table 1, Figure 1).

Seventy-one *I. cookei* ticks (n = 41 pools) and two *I. scapularis* (n = 2 pools) were removed and tested from 29 woodchucks. Two strains of POWV-1 (46-001 and 63-002) were isolated from engorged *I. cookei* ticks. POWV-positive ticks were collected from woodchucks trapped in Jefferson and Orange Counties (Figure 1). Both isolates were from tick pools consisting of an individual replete female removed from two different hosts. No other ticks were positive, including an engorged nymph *I. cookei* tick removed from one woodchuck with a positive adult female tick.

### Genomic sequencing of recent POWV-1 NYS isolates

**Figure 2.**
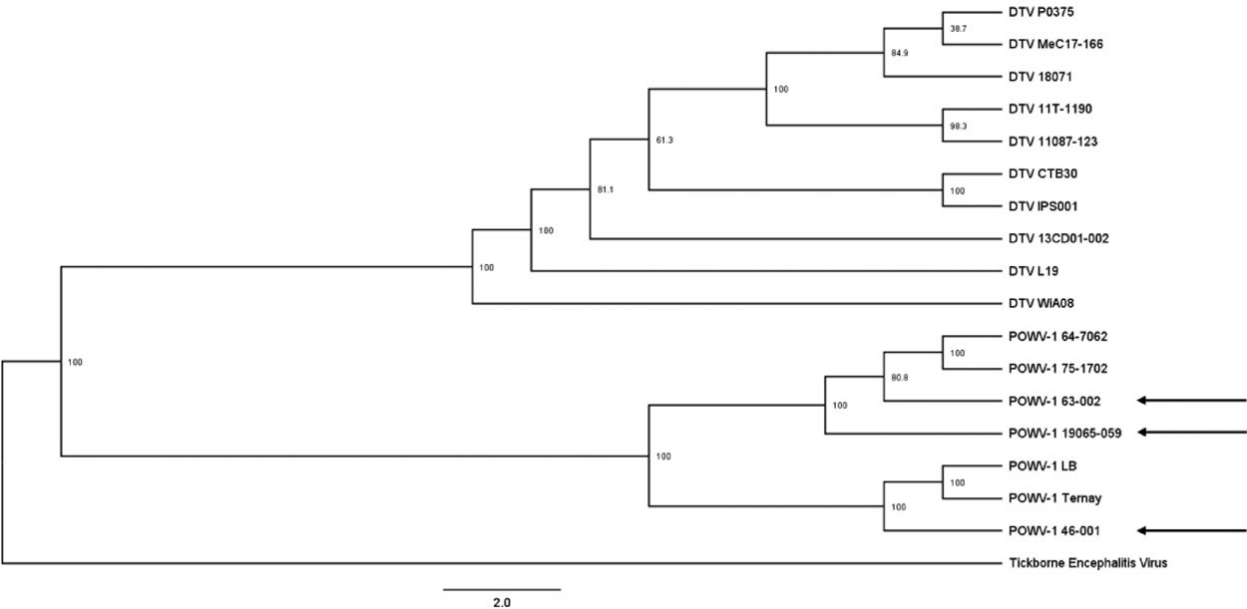
Maximum likelihood phylogeny of Powassan virus (POWV-1) and deer tick virus (DTV) based on full genome nucleotide sequences with tickborne encephalitis virus as an outgroup. Bootstrap values are displayed at each node (range: 38.9-100). Recent New York State (NYS) isolates (arrows); POWV-1 46-001, POWV-1 63-002, and POWV-1 19065–059 cluster with historic POWV-1 isolates.

To assess the genetic variation of recent NYS POWV-1 isolates: POWV-1 46-001, POWV-1 63-002, and POWV-1 19065-059, full genome sequencing was completed, and consensus sequences were compared to historic POWV-1 and DTV isolates from North America and Russia. All recent NYS POWV-1 isolates cluster within lineage 1 (Figure 1). However, while POWV-1 63–002 and POWV-1 19065–059 cluster closely with other POWV-1 isolates from NYS, POWV-1 46–001 shares more genetic similarity to POWV-1 LB and POWV-1 Ternay (Figure 2).

Investigation of individual nucleotide and amino acid changes revealed genetic divergence between the three recent isolates. Mean pairwise genetic distance on the nucleotide level was 93.0-96.6%. Of the 25 identified amino acid substitutions, 7 were unique to POWV-1 46-001, 6 were unique to POWV-1 63-002, and 9 were unique to POWV-1 19065–059 (Table 2). In addition, POWV-1 46–001 displayed 11 amino acid substitutions that are shared with only POWV-1 LB (data not shown). POWV-1 46–001 also shares 8 substitutions with POWV-1 LB and all known DTVs (data not shown) and exhibits 1 substitution that is only found in previously sequenced DTVs or a “lineage-2-like substitution” (S1660N). POWV-1 63–002 possesses the most lineage-2-like substitutions (n = 3, R687 K, S1339P, A1416 V) and shares 1 additional substitution with POWV-1 19065-059. Despite being isolated from an *I. scapularis*, POWV-1 19065–059 displayed no unique lineage-2-like substitutions. In addition, no unique substitutions were shared among all three recent POWV-1 isolates. Mutations were dispersed throughout the POWV-1 genome, and strain-specific substitutions were identified in all viral proteins excluding the capsid (Table 2).

**Table 2.** Amino acid substitutions for recent Powassan virus (POWV-1) isolates from New York State (NYS) as compared to all available sequenced POWV-1 and deer tick virus (DTV) isolates. Unique substitutions are **bolded**. Lineage-2-like substitutions are highlighted in light grey.

Genome Position	AA Position	POWV-1 46–001	POWV-1 63–002	POWV-1 19065–059	POWV-1 64–7062	DTV 18071–054
PrM	259	**GLY**	SER	SER	SER	SER
PrM	262	**VAL**	ALA	ALA	ALA	ALA
PrM	265	THR	THR	**ILE**	THR	THR
E	312	THR	THR	**SER**	THR	THR
E	366	**PRO**	ALA	ALA	ALA	ALA
E	404	**ARG**	LYS	LYS	LYS	LYS
E	405	ALA	**VAL**	ALA	ALA	ALA
E	421	**ALA**	VAL	VAL	VAL	VAL
E	439	LYS	LYS	**ARG**	LYS	LYS
E	453	ARG	ARG	**GLN**	ARG	ASN
E	687	ARG	LYS	ARG	ARG	LYS
NS1	1004	ALA	**PRO**	ALA	ALA	ALA
NS2a	1292	ARG	ARG	**LYS**	ARG	ARG
NS2a	1303	VAL	**ILE**	VAL	VAL	VAL
NS2a	1339	SER	PRO	SER	SER	PRO
NS2b	1416	VAL	ALA	VAL	VAL	ALA
NS3	1660	SER	ASN	ASN	ASN	SER
NS3	1930	ILE	**VAL**	ILE	ILE	ILE
NS4a	2121	**SER**	GLY	GLY	GLY	GLY
NS4b	2286	VAL	**ALA**	**ALA**	VAL	VAL
NS4b	2315	MET	**VAL**	MET	MET	MET
NS4b	2415	ILE	ILE	**VAL**	ILE	ILE
NS5	2538	ALA	ALA	**SER**	ALA	ALA
NS5	2692	**VAL**	ALA	ALA	ALA	ALA
NS5	3018	ALA	ALA	**SER**	ALA	ALA

### NYS POWV-1 isolates display variable infectivity and replicative fitness in arthropod hosts

**Figure 3.**
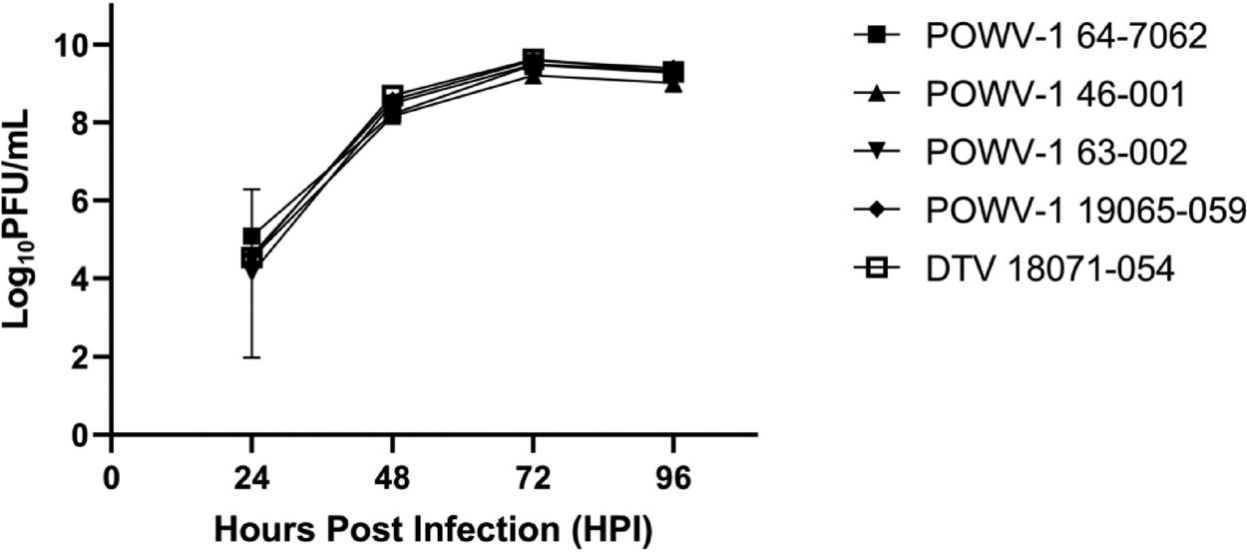
Growth kinetics of Powassan virus (POWV-1) and deer tick virus (DTV) in baby hamster kidney (BHK-21) cells. Replication in BHK-21 is similar among all isolates. Data points represent mean +/- SEM (n = 3 per strain). Two-way ANOVA and Tukey’s multiple comparisons test (*p *= 0.8261).

To evaluate the replicative fitness of recent NYS POWV-1 isolates in representative hosts we assessed growth kinetics *in vitro* and *in vivo*. Isolates were grown in baby hamster kidney cells (BHK-21) and *I. scapularis* nymphs to represent mammalian and invertebrate hosts, respectively. Replication kinetics within BHK-21 cells displayed no significant differences in viral titre over time or between strains (Figure 3; Two-way ANOVA [*p *= 0.826] and Tukey’s multiple comparisons test, [*p *> 0.05]).

**Figure 4.**
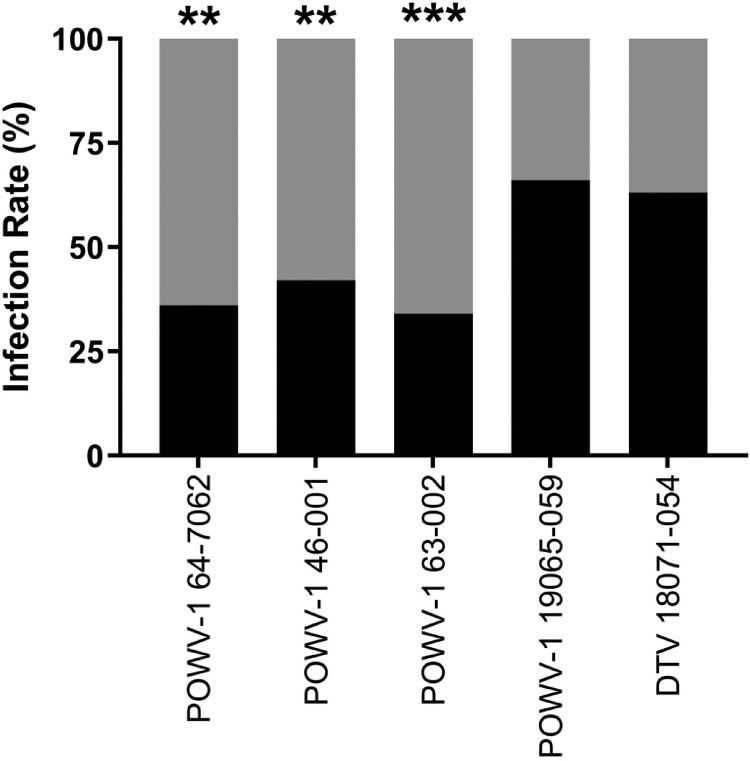
Overall infection rates of New York State (NYS) Powassan virus (POWV-1) and deer tick virus (DTV) isolates in *Ixodes scapularis* nymphs following immersion. Black shading represents the percent of infected ticks with POWV-1 or DTV and gray shading represent the percent of uninfected ticks. Infected ticks across all timepoints (7-,14-,21-, and 28-days post infection) were combined to calculate overall percentage of POWV-infected ticks (n = 80/strain for POWV-1 64–7062 and POWV-1 46-001, n = 90/strain for POWV-1 63-002, POWV-1 19065-059, and DTV 18071-054). POWV-1 64-7062, POWV-1 46-001, and POWV-1 63–002 displayed significantly lower infection rates compared to POWV-1 19065–059 and DTV 18071–054 (chi-squared test, **p < 0.01, ***p < 0.001).

To assess viral growth in the invertebrate host, *I. scapularis* nymphs were infected by immersion and harvested at 7-, 14-, 21-, and 28-days post infection (DPI) [36, 37]. Two separate experiments were conducted and combined as viral titres within individual ticks were not significantly different between different experiments (t-test, *p *> 0.05). Infection rates from all timepoints were combined and found to be significantly different between the *I. cookei* and *I. scapularis*-derived isolates (Figure 4; chi-squared test, *p *< 0.01). POWV-1 64-7062, POWV-1 46-001, and POWV-1 63–002 immersion resulted in infection of 36.3%, 42.5%, and 30.0%, respectively, of experimentally infected *I. scapularis,* while DTV 18071–054 and POWV-1 19065–059 immersion resulted in infection rates of 57.0% and 59.0%, respectively.

**Figure 5.**
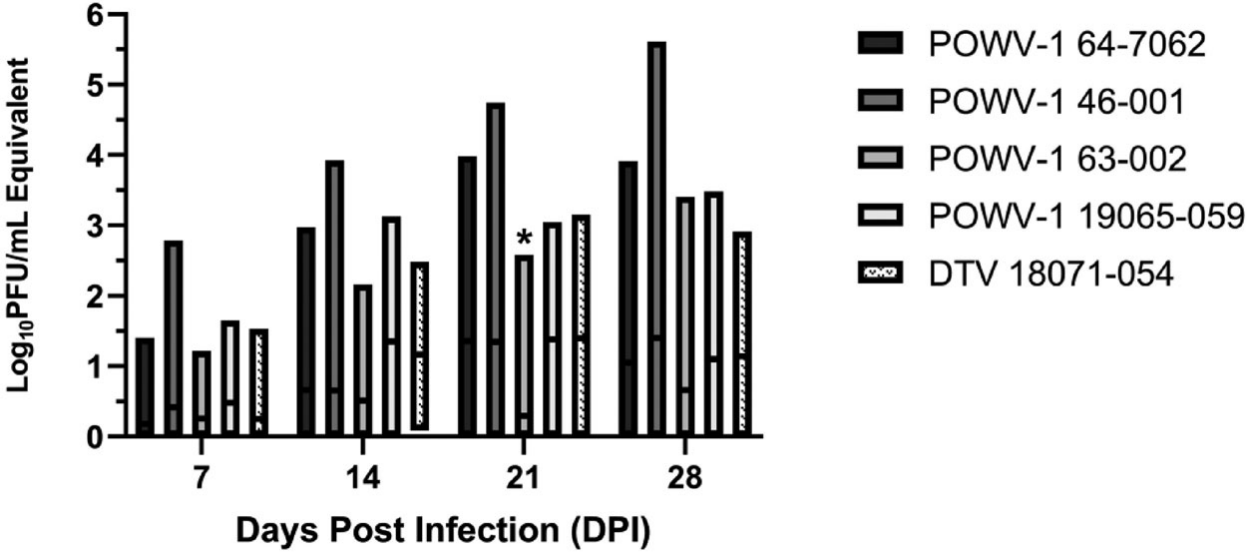
Growth kinetics of New York State (NYS) Powassan virus (POWV-1) and deer tick virus (DTV) isolates in *Ixodes scapularis* nymphs following immersion. Individual data include two separate, statistically equivalent experiments (t-test, *p *> 0.05, n = 4-19). Time (DPI) significantly influenced viral load (two-way ANOVA, *p* < 0.0001). Pairwise comparisons indicated statistical equivalence of viral load among strains, with the exception of POWV-1 63-002, which was significantly lower than all other strains at 21 DPI (two-way ANOVA, Tukey’s multiple comparisons, *p *< 0.017).

Viral titres within individual ticks were variable, ranging from 0.5–5.6 log_10_PFU/mL equivalents (Figure 5). POWV-1 19065–059 reached peak viral load the earliest while POWV-1 46–001 continued to grow to the highest titres by day 28. Of note, POWV-1 63–002 exhibited the most attenuated growth, reaching just over 1.0 log_10_PFU/mL across all timepoints. Although time significantly influenced viral load (two-way ANOVA, *p *< 0.0001), differences resulted primarily from variability in individual ticks, which precluded the capacity to identify a significant effect of strains despite variable trends (two-way ANOVA, *p *= 0.065). While mean viral titres were higher for DTV 18071–054 and POWV-1 19065–059 at 14 and 21 DPI, the only statistically significant pairwise differences identified among strains were between POWV-1 63–002 and all other strains at 21 DPI (Tukey’s multiple comparisons test, *p *< 0.02). Despite variability, infectivity and viral replication data together suggest *I. scapularis*-derived strains (DTV 18071–054 and POWV-1 19065-059) possess higher replicative fitness levels in *I. scapularis* relative to *I. cookei*-derived strains.

## Discussion

This study reports the first isolations of POWV-1 in NYS since 1975. Additionally, this is the first known isolation and characterization of POWV-1 from *I. scapularis*. While POWV-1 is primarily maintained by *I. cookei,* it has previously been isolated from non-*Ixodes spp.* including *H. longicornus* in Russia [2, 18]. To date, DTV has been detected and isolated from *I. scapularis* and *Dermacentor spp.* in the US [17]. The isolation of POWV-1 from *I. scapularis* likely represents a spillover event of POWV-1 into a novel enzootic cycle. In addition to isolation of POWV-1 from a novel invertebrate host, we also report the first isolations of POWV-1 from new locations in Central NYS. These isolations offer insight into the current genetic and phenotypic diversity of POWV-1 in these regions.

All three recent POWV-1 isolates displayed unique genotypes and phenotypes that were distinct from each other, historic POWV-1 isolates, and DTV isolates. These data suggest long-term maintenance of POWV-1 in distinct ecological and geographic foci facilitates genetic distinction, potentially due to the limited migratory range of mammalian hosts and tick vectors. Previous work has detailed a similar stable maintenance of highly homogenous DTV populations in discrete foci [4, 5, 12, 21]. For example, Brackney et al. [12] found homogenous DTV populations from Spooner and Hayward, Wisconsin sampled in 1997 and 2007-2008. Similarly, Midwestern and Northeastern US DTV isolates share 90% sequence identity but ∼99% sequence identity within clades [4, 5, 21]. Similar studies on the closely related flavivirus, tick-borne encephalitis virus (TBEV) demonstrate stable maintenance of discrete TBEV foci spatially and temporally [38]. The westward expansion and establishment of TBEV into Asia from Europe resulted in genetic divergence now recognized as three subtypes: European, Siberian, and far-eastern. Our data demonstrate that POWV-1 46-001, isolated in northern NYS, was more closely related to historic POWV-1 isolates that were detected in northern NYS and Canada than other recent POWV-1 isolates derived from central and southern regions of NYS. Overall, these data further support the paradigm that POWV is maintained stably over time and space. Further, no shared amino acid substitutions were identified among the three recent isolates relative to historic POWV-1 and DTV isolates, suggesting discrete evolutionary histories.

Recent NYS POWV-1 isolates contain multiple unique non-synonymous mutations, and this genotypic variability correlates to phenotypic variability in *I. scapularis*. Many of the identified non-synonymous changes do not result in changes in amino acid size, charge, or polarity. This would suggest a limited role in affecting viral fitness, but some mutations may act in combination to result in phenotypic changes [39]. Additionally, studies on other pathogenic flaviviruses (i.e. Zika virus, West Nile virus, and dengue virus) have demonstrated the potential for single non-synonymous mutations to result in significant phenotypic changes [40–42]. Future work with infectious clones is needed to isolate mutations of interest to measure direct effects on viral phenotype. Despite this variability, some important lineage and vector-specific trends were evident. Both *I. cookei*-derived strains displayed evidence of attenuation in *I. scapularis.* In addition, despite the lack of novel lineage-2-like mutations identified in the *I. scapularis*-derived POWV-1 19065-059, significantly higher infection rates in *I. scapularis*, similar to those identified for DTV, were measured relative to *I. cookei*-derived isolates. The increased infectivity and fitness of POWV-1 19065–059 in *I. scapularis* suggests this strain may have further adapted to *I. scapularis*. While the mechanistic basis for this is unclear, the unique substitutions identified in structural and nonstructural genes offer potential targets for studying species-specific fitness.

Experimental infections with prototype POWV-1 have shown that *I. scapularis* is a competent vector that could play a role in maintenance and transmission of POWV-1 [43, 44]. In addition, infections of *Amblyomma spp*. and *Dermacentor spp*. with DTV demonstrate competence despite decreased viral loads relative to *I. scapularis* [45], and *D. andersoni ex vivo* cultures have been shown to support POWV-1 replication [46]. While these studies were completed with individual strains that may not be representative of phenotypic diversity in nature, they suggest species-specific competence may not be a primary barrier to ecological and evolutionary distinction of POWV. Despite this, our data reveal host factor differences in *I. cookei* and *I. scapularis* do likely contribute to lineage separation and that evolution could contribute to further expansion. The limitations of infection by immersion should be considered as this method excludes the intake of a bloodmeal which induces physiological changes within the tick that could influence vector competence [47]. Further studies with POWV animal models could clarify strain-specific differences in transmissibility and disease in the tick host. Additionally, limitations of measuring phenotypes in BHK-21 cell lines, which are not completely representative of viral kinetics within mammalian hosts in nature, should be considered. While our experimental infections displayed no significant differences in replicative fitness in BHK-21, these cells lack a competent interferon system which is a primary antiviral response in mammalian hosts [48]. To better understand strain-specific phenotypes in mammalian hosts studies using IFN-competent systems or animal models are needed.

The detection of POWV-1 in *I. scapularis* demonstrates the importance of maintaining robust surveillance of tick-borne viruses that can effectively track the establishment of POWV-1 and DTV in novel locations and hosts. Increased POWV-1 prevalence and diversification in the Northeast US in combination with the expansion of numerous competent vector species could facilitate further spillover events into novel enzootic cycles, particularly because many tick species feed on similar mammalian hosts [47]. Future studies investigating the genetic correlates of strain-specific fitness and the capacity for further adaptation of POWV to novel invertebrate hosts could provide insight into the potential role of genetic diversification in driving expanded transmission and disease.
